# Improvement of Refractory Cutaneous Ulcers and Pulmonary Arterial Hypertension Following Rituximab and Mycophenolate Mofetil Therapy in Anti-melanoma Differentiation-Associated Gene 5 (Anti-MDA5) Antibody-Positive Dermatomyositis

**DOI:** 10.7759/cureus.111928

**Published:** 2026-07-01

**Authors:** Takuya Yasuda, Tatsuyoshi Fujii, Noboru Hagino

**Affiliations:** 1 Division of Rheumatology, Teikyo University Medical Center in Chiba, Ichihara, JPN; 2 Department of General Internal Medicine, Akita Kousei Medical Center, Akita, JPN

**Keywords:** anti-mda5 dermatomyositis, connective tissue disease, cutaneous ulcers, mycophenolate mofetil, pregnancy, pulmonary arterial hypertension, rituximab, vasculopathy

## Abstract

Anti-melanoma differentiation-associated gene 5 (anti-MDA5) antibody-positive dermatomyositis (DM) can manifest with a severe vasculopathic phenotype. We report a case of anti-MDA5 DM in a woman in her early 30s complicated by refractory cutaneous ulcers and pulmonary arterial hypertension (PAH). Despite the escalation of immunosuppressive therapy with glucocorticoids, tacrolimus, intravenous immunoglobulin, and cyclophosphamide, the cutaneous ulcers remained refractory. A progressive decline in diffusing capacity of the lung for carbon monoxide raised suspicion of PAH, which was subsequently confirmed by right heart catheterization. Consequently, combination therapy with mycophenolate mofetil (MMF) and rituximab (RTX) was initiated alongside bosentan. This regimen resulted in the prompt resolution of ulcers and improvement of PAH. Bosentan was discontinued, and azathioprine was initiated to maintain remission, allowing for a successful pregnancy and delivery of a healthy infant. This case suggests a potential role for an MMF- and RTX-containing combination regimen in refractory cutaneous ulcers and PAH associated with anti-MDA5 DM.

## Introduction

Anti-melanoma differentiation-associated gene 5 (anti-MDA5) antibody-positive dermatomyositis (DM) is associated with rapidly progressive interstitial lung disease (ILD) and severe vasculopathy [1,2]. Pulmonary hypertension (PH) is defined as a mean pulmonary artery pressure >20 mmHg measured by right heart catheterization [3], and precapillary PH is classified as pulmonary arterial hypertension (PAH) [4]. Among the recognized clinical subgroups of anti-MDA5 antibody-positive DM, a vasculopathic phenotype has been described [5].

Connective tissue diseases (CTDs) are an important cause of PAH in Asia [3]. Whereas systemic sclerosis is the predominant CTD associated with PAH in Western cohorts [6], systemic lupus erythematosus and mixed CTD account for a relatively larger proportion of CTD-associated PAH (CTD-PAH) cases in Asian populations [7]. Although PAH has been reported in DM [8], its occurrence in anti-MDA5 antibody-positive DM remains extremely rare. We report a case of anti-MDA5 antibody-positive DM complicated by PAH and severe cutaneous vasculopathy.

## Case presentation

A Japanese woman in her early 30s was referred to our hospital for further management of anti-MDA5 antibody-positive clinically amyopathic DM with persistent cutaneous manifestations. One month before referral, chest computed tomography (CT) at a previous hospital showed minimal ILD. At that stage, facial erythema and pruritic scaly lesions of the scalp persisted despite treatment with topical corticosteroids and several courses of oral antibiotics; topical corticosteroids had provided only transient improvement. Her respiratory status was stable, and muscle strength was normal, with a Manual Muscle Testing-8 (MMT-8) score of 150/150 according to the Medical Research Council grading. Because there were no clinical or radiological features suggestive of rapidly progressive ILD, aggressive immunosuppressive therapy was not initiated.

At the initial visit, she had Gottron's papules, periungual erythema, mechanic's hands, and scaly alopecia, without heliotrope rash or muscle weakness. During outpatient follow-up, her cutaneous manifestations progressed, with painful digital ulcers, worsening palmar erythema (Figure 1), and dysphagia, although she did not report significant worsening of dyspnea. Follow-up CT showed increased density and mild extension of subpleural interstitial infiltrates, consistent with the progression of nonspecific interstitial pneumonia (NSIP)-type ILD. There were no radiological features suggestive of infection, organizing pneumonia, or alternative pathology. Malignancy screening, including contrast-enhanced CT, upper and lower gastrointestinal endoscopy, cervical cytology, and mammography, showed no evidence of malignancy.

**Figure 1:**
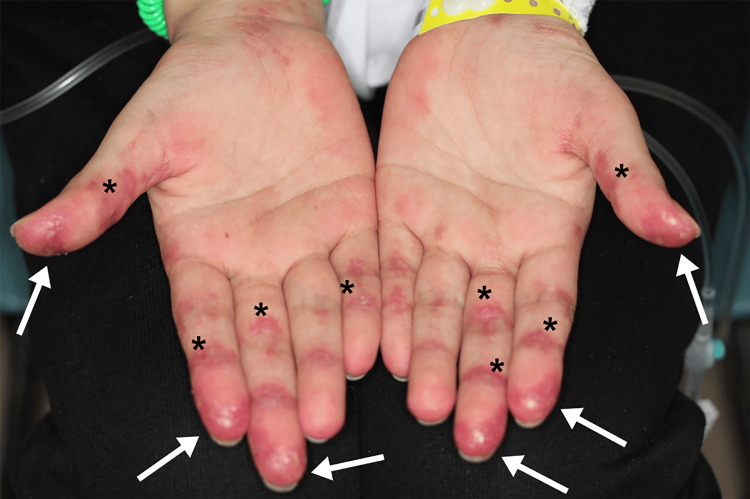
Cutaneous manifestations at the time of exacerbation (month 3) Multiple erythematous papules and plaques are observed on the volar aspect of the fingers (asterisks), accompanied by painful digital ulcers on the fingertips (arrows).

Investigations

At the initial presentation, laboratory testing showed elevated ferritin, mildly elevated creatine kinase (CK) and lactate dehydrogenase (LDH), and a positive anti-MDA5 antibody result. The results of the initial laboratory evaluation are presented in Table 1. Serum ferritin was measured as a marker of systemic inflammation and disease activity in anti-MDA5 antibody-positive DM.

**Table 1:** Key laboratory findings at the initial presentation and autoantibody profile

Laboratory parameter	Result	Reference range	Unit
Hematology			
White blood cell count	3,900	3,500-8,100	/µL
Hemoglobin	12.3	11.3-15.2	g/dL
Platelet count	191,000	130,000-369,000	/µL
Biochemistry and inflammatory markers			
Lactate dehydrogenase	265	115-245	U/L
Creatine kinase	236	45-163	U/L
Serum creatinine	0.50	0.47-0.79	mg/dL
C-reactive protein	<0.3	<0.3	mg/dL
Erythrocyte sedimentation rate	27	3-15	mm/h
Ferritin	145.4	3.6-114	ng/mL
Autoantibody profile			
Anti-melanoma differentiation-associated gene 5 antibody	157	0-32	index
Anti-topoisomerase I antibody	<1	0-10	U/mL
Anti-U1 ribonucleoprotein antibody	2	0-10	U/mL
Anti-Sjögren's syndrome-related antigen A antibody	<1	0-10	U/mL
Anti-Sjögren's syndrome-related antigen B antibody	<1	0-10	U/mL
Anti-Jo-1 antibody	<1	0-10	U/mL

Overlap CTDs were excluded by serological and clinical evaluation. The relevant autoantibody profile is shown in Table 1. She had no clinical features suggestive of systemic sclerosis, including Raynaud's phenomenon, sclerodactyly, nailfold capillary abnormalities, or telangiectasia.

During disease exacerbation, pulmonary function tests showed a marked decline in diffusing capacity of the lung for carbon monoxide (DLCO), from 76.1% at baseline to 34.5% and then to 28.4% one month later. In contrast, forced vital capacity (FVC) was relatively preserved: 2.75 L (87.6% predicted) when DLCO declined to 42% and 2.84 L (90.2% predicted) when DLCO further decreased to 28%. Chest CT showed enlargement of the main pulmonary artery trunk (Figure 2), which appeared disproportionate to the extent of ILD. Transthoracic echocardiography at month 5 showed a tricuspid regurgitation pressure gradient (TRPG) of 37.9 mmHg, raising suspicion of PH and prompting referral to the cardiology department for further evaluation. CT pulmonary angiography showed no pulmonary embolism or chronic thromboembolic disease. Right heart catheterization at month 6 confirmed precapillary PH, with a mean pulmonary artery pressure of 31 mmHg, pulmonary artery wedge pressure of 9 mmHg, cardiac output of 6.36 L/min, and pulmonary vascular resistance of 3.5 Wood units (WU).

**Figure 2:**
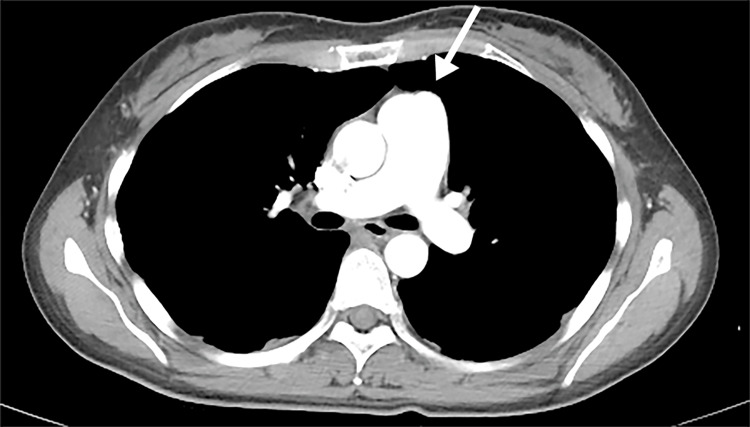
Radiological findings on chest computed tomography Chest computed tomography reveals an enlargement of the main pulmonary artery trunk (arrow), which appeared disproportionate to the extent of the interstitial lung disease.

During the clinical course, thrombocytopenia and elevated LDH raised suspicion of thrombotic microangiopathy (TMA).

Treatment

Phase 1: Initial Refractory Phase

At the initial presentation, disease severity was considered limited because chest CT showed only minimal subpleural interstitial changes, oxygen saturation was preserved, muscle strength was normal, and CK and ferritin were only mildly elevated. Prednisolone 20 mg/day and tacrolimus 0.5 mg/day were therefore started as a stepwise therapeutic approach. Tacrolimus was temporarily discontinued owing to hepatotoxicity but was later reintroduced. At month 3, because of the progression of ILD and cutaneous ulcers, she received methylprednisolone pulse therapy and intravenous immunoglobulin (IVIG) (20 g/day for five days).

Phase 2: Intensification and Complications

At approximately month 4, intravenous cyclophosphamide was initiated with leuprorelin for ovarian protection. She received three intravenous doses at approximately two-week intervals: 500 mg for the first dose and 750 mg for the second and third doses. Short-term oral cyclophosphamide, 250 mg daily for three days, was also administered. Despite this treatment, DLCO continued to decline, and the cutaneous ulcers worsened. She developed ulcerative erythema on the left upper eyelid and deep ulcers on the dorsal aspect of the hands (Figure 3). The course was complicated by *Staphylococcus *tenosynovitis requiring surgical drainage at month 5.

**Figure 3:**
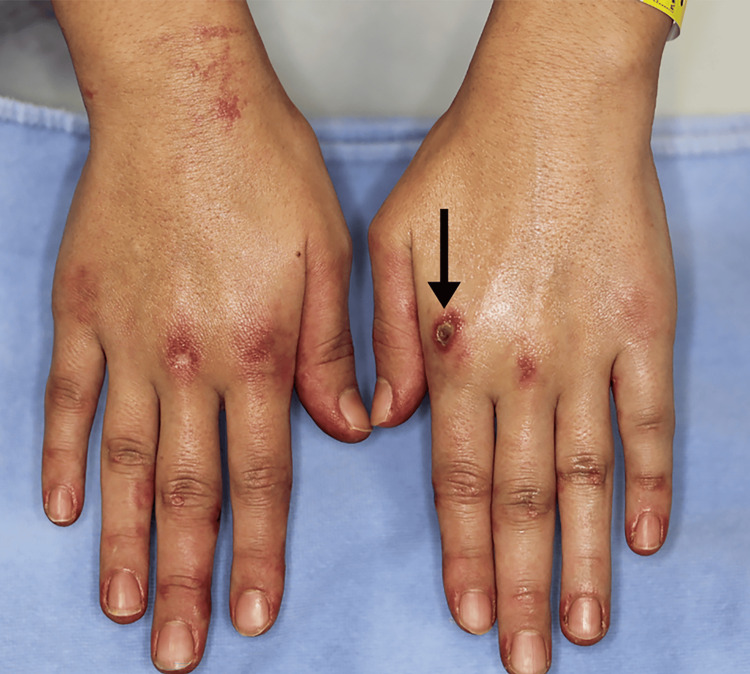
Deep cutaneous ulcers on the hands A deep, refractory ulcer (arrow) is seen on the dorsal aspect of the hand. These lesions were resistant to initial aggressive immunosuppressive therapy, including steroid pulse therapy and intravenous immunoglobulin.

Laboratory abnormalities suggestive of tacrolimus-associated TMA subsequently appeared, with a platelet count of 118,000/µL, LDH of 397 U/L, and serum creatinine of 0.85 mg/dL. No schistocytes were identified on peripheral blood smear, and ADAMTS13 (a disintegrin and metalloproteinase with a thrombospondin type 1 motif, member 13) activity was not measured. These abnormalities improved after tacrolimus discontinuation. For newly diagnosed PAH and severe digital vasculopathy, prostaglandin E1 infusion and bosentan were initiated. Bosentan was started at 125 mg/day and increased to 250 mg/day after one month.

Phase 3: Rescue With Rituximab (RTX) and Mycophenolate Mofetil (MMF)

Six months after referral, given the refractory nature of the disease involving ILD, vasculopathy, and PAH, we initiated a salvage regimen informed by the "rituxilup" protocol described by Condon et al. [9], which combines RTX with MMF as a steroid-minimizing strategy. This approach was selected to achieve B-cell-targeted immunomodulation while avoiding further escalation of glucocorticoid exposure. She received four weekly doses of RTX (500 mg), combined with MMF. At RTX initiation, prednisolone was 7 mg/day and was gradually tapered to 6 mg/day during the induction. MMF was titrated to 1 g/day, but further dose escalation was avoided because alopecia remained a significant cosmetic concern for the patient.

Outcome and follow-up

After RTX and MMF induction, the digital, dorsal hand, and eyelid ulcers gradually healed, and the dysphagia resolved. The ILD remained stable during follow-up, and pulmonary hemodynamics improved (Figure 4). Two months after RTX initiation, repeat right heart catheterization showed normalization of hemodynamics at rest, with a mean pulmonary arterial pressure of 10 mmHg and pulmonary vascular resistance of 1.02 WU, with normal hemodynamic responses during exercise. She was initially maintained on MMF 1 g/day. Around year 2, in anticipation of a future pregnancy, MMF was gradually tapered, while azathioprine was introduced and titrated up to 75 mg/day over two months. Bosentan was also tapered and subsequently discontinued during this period. Serial anti-MDA5 antibody measurements were initiated after clinical stabilization, when bosentan tapering had begun. All subsequent measurements were negative. However, because antibody levels had not been serially assessed during the active phase, the relationship between changes in anti-MDA5 antibody levels and clinical improvement could not be evaluated. Long-term follow-up using transthoracic echocardiography between years 2 and 5 after the initial presentation confirmed that the TRPG remained stable between 11.7 and 14.7 mmHg, indicating sustained remission of PAH without pulmonary vasodilators. She remained in remission on azathioprine monotherapy. Seven years after the initial presentation, she conceived naturally and delivered a healthy infant.

**Figure 4:**
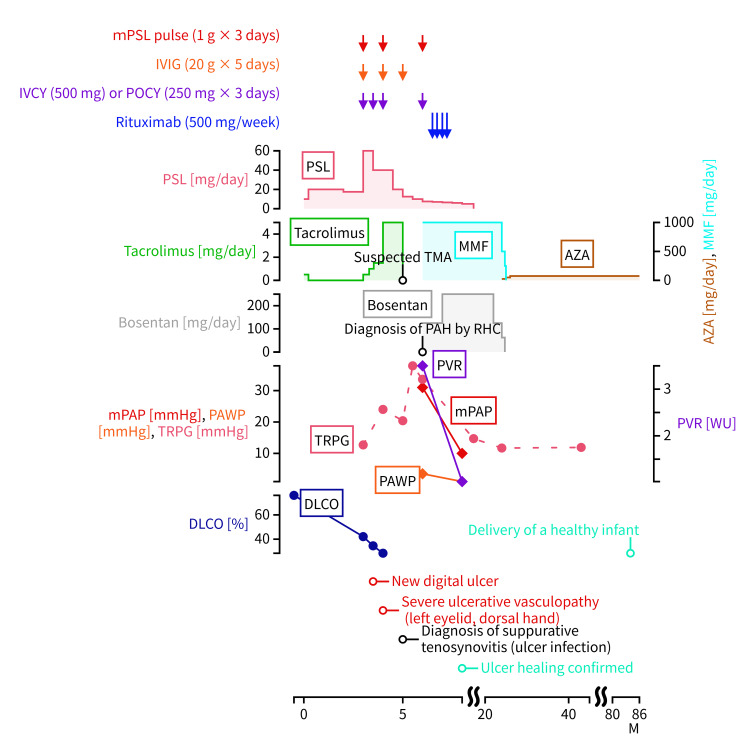
Clinical course and response to treatment mPAP: mean pulmonary artery pressure; PVR: pulmonary vascular resistance; PAWP: pulmonary artery wedge pressure; RHC: right heart catheterization; TRPG: tricuspid regurgitation pressure gradient; DLCO: diffusing capacity of the lung for carbon monoxide; MMF: mycophenolate mofetil; PAH: pulmonary arterial hypertension; PSL: prednisolone; mPSL: methylprednisolone; IVIG: intravenous immunoglobulin; IVCY: intravenous cyclophosphamide; POCY: peroral cyclophosphamide; AZA: azathioprine; TMA: thrombotic microangiopathy; WU: Wood units The timeline illustrates changes in pulmonary hemodynamics and lung function alongside therapeutic interventions. It includes mPAP, PVR, and PAWP measured by RHC, TRPG monitored by transthoracic echocardiography, and DLCO. The graph demonstrates the resolution of refractory complications following the initiation of the rescue regimen consisting of rituximab and MMF. Long-term follow-up from year 2 to year 5 showed that the TRPG remained stable within the normal range of 11.7-14.7 mmHg, indicating sustained remission of PAH even after bosentan discontinuation. This figure was created manually by Takuya Yasuda from the patient's clinical data using LafLabo (LafLabo Co., Ltd., Osaka, Japan), a web-based tool. No generative artificial intelligence or artificial intelligence-assisted image generation tools were used.

## Discussion

We report a case of anti-MDA5 antibody-positive DM complicated by severe cutaneous vasculopathy and PAH. Although the disease was refractory to glucocorticoids, cyclophosphamide, tacrolimus, and IVIG, combination therapy with RTX and MMF was associated with the resolution of cutaneous ulcers and the normalization of pulmonary hemodynamics.

Anti-MDA5 antibody-positive DM carries a high risk of rapidly progressive ILD, with reported mortality rates of 30-50% in severe cases, particularly in Asian cohorts [10]. However, this case was notable for a vasculopathic phenotype. Cluster 3 anti-MDA5 antibody-positive DM, as described by Allenbach et al. [5], is characterized by cutaneous vasculopathy, including Raynaud's phenomenon, skin ulcers, digital necrosis, and calcinosis. Our patient had classic DM rashes, refractory ulcers, elevated CK, and ILD, consistent with this phenotype.

PAH is a rare complication of idiopathic inflammatory myopathies. In the French PH registry, only three cases of PAH associated with idiopathic inflammatory myopathy were identified among 5,223 patients with PH [8]. DM subtype, cutaneous manifestations, peripheral microangiopathy, and anti-SSA antibodies have been suggested as possible associated factors [8]. These findings support the hypothesis that systemic vasculopathy and microvascular injury may contribute to pulmonary vascular involvement in idiopathic inflammatory myopathies.

In this case, PH was interpreted as CTD-PAH rather than PH secondary to ILD, because ILD was limited, FVC was preserved, DLCO declined disproportionately, and right heart catheterization confirmed precapillary PH. CT pulmonary angiography excluded pulmonary embolism and chronic thromboembolic disease. Because ILD represented a cardiopulmonary comorbidity, initial PAH monotherapy was considered appropriate under current European Society of Cardiology (ESC)/European Respiratory Society (ERS) guideline recommendations [6]. Bosentan was selected because endothelin receptor antagonists may also be beneficial for CTD-PAH with digital vasculopathy [11]. Anticoagulation was not initiated because thromboembolic disease was excluded and the suspected TMA raised concern about bleeding risk.

For ILD associated with anti-MDA5 antibody-positive DM, early triple immunosuppressive therapy with high-dose glucocorticoids, tacrolimus, and cyclophosphamide has been shown to improve survival [10]. Janus kinase inhibitors have recently emerged as a potential option, particularly for rapidly progressive ILD, but evidence in cases complicated by PAH or severe vasculopathy remains limited. There is no established standard treatment for refractory cutaneous ulcers in anti-MDA5 antibody-positive DM. IVIG is the only therapy with efficacy clearly demonstrated in a randomized controlled trial for refractory DM [12], whereas case reports have described the successful use of MMF or RTX for anti-MDA5 antibody-positive cutaneous ulcers [13,14]. Clottu et al. reported an anti-MDA5 antibody-positive DM case with refractory mucocutaneous lesions, periungual inflammation, digital ulcers, and necrosis. The mucocutaneous manifestations improved substantially after RTX despite prior treatment with glucocorticoids, IVIG, MMF, cyclosporine, and cyclophosphamide [14]. However, digital arterial occlusion and fingertip necrosis subsequently developed, suggesting that RTX may improve inflammatory mucocutaneous disease without necessarily preventing the progression of severe vasculopathy. In contrast, our patient had both severe cutaneous vasculopathy and right heart catheterization-confirmed PAH, with improvement of the cutaneous lesions and pulmonary hemodynamics after combined RTX, MMF, and bosentan therapy. Nevertheless, because several therapies were administered concomitantly, the specific contribution of RTX and MMF cannot be determined. In this case, RTX and MMF were selected as rescue immunosuppressive therapy for refractory systemic vasculopathic disease, with the expectation that immune-mediated vascular inflammation might also contribute to the coexisting PAH.

Immunosuppressive therapy may improve CTD-PAH in selected non-systemic sclerosis CTDs. Sanchez et al. reported that, among patients with CTD-PAH treated with immunosuppression, responses were observed in systemic lupus erythematosus and mixed CTD, but not in systemic sclerosis [15]. In our case, DLCO continued to decline despite glucocorticoids, cyclophosphamide, and tacrolimus, whereas pulmonary hemodynamics subsequently improved after the initiation of RTX, MMF, and bosentan. The temporal association suggests that RTX and MMF may have contributed to the improvement; however, a direct effect of bosentan and delayed effects of the preceding immunosuppressive therapies, including glucocorticoids, cyclophosphamide, and IVIG, cannot be excluded. The sustained remission after bosentan discontinuation suggests that immune-mediated vasculopathy may have contributed to the pulmonary vascular disease, but does not establish that the improvement was attributable specifically to RTX and MMF.

Tacrolimus was discontinued because of TMA-like laboratory abnormalities, including thrombocytopenia and elevated LDH. Recent evidence suggests that calcineurin inhibition can promote endothelial activation, increased vascular resistance, and adverse cardiovascular effects [16]. The improvement in laboratory abnormalities after tacrolimus discontinuation supports a drug-associated vascular complication in this case.

Cyclophosphamide is highly gonadotoxic, with potential adverse effects on ovarian function. In young women requiring urgent cytotoxic therapy, fertility preservation options may be limited by the need for prompt disease control. Although oocyte or embryo cryopreservation is an established approach, it may not always be feasible in urgent clinical settings because it requires ovarian stimulation and procedural preparation [17]. In this case, leuprorelin was administered as a practical ovarian-protective measure before cyclophosphamide initiation [17,18].

## Conclusions

This case suggests that PAH and severe cutaneous vasculopathy associated with anti-MDA5 antibody-positive DM may be reversible following intensive immunosuppressive and PAH-targeted therapy, including RTX, MMF, and bosentan. It also highlights the importance of considering PAH when DLCO declines disproportionately to the extent of ILD. Given the single-case nature of this report and the use of multiple concomitant therapies, the findings cannot be generalized to all patients with anti-MDA5 antibody-positive DM, and the specific contribution of RTX and MMF cannot be determined.
